# Identification of miRNAs Involved in *Bacillus velezensis* FZB42-Activated Induced Systemic Resistance in Maize

**DOI:** 10.3390/ijms20205057

**Published:** 2019-10-12

**Authors:** Shanshan Xie, Hengguo Yu, Enze Li, Yu Wang, Juan Liu, Haiyang Jiang

**Affiliations:** 1 The National Key Engineering Lab of Crop Stress Resistance Breeding, School of Life Sciences, Anhui Agricultural University, Hefei 230036, China; xssflora871216@126.com (S.X.); 15966637205@163.com (H.Y.); wangyu20180712@163.com (Y.W.); liujuanxf@126.com (J.L.); 2 School of Horticulture, Anhui Agricultural University, Hefei 230036, China

**Keywords:** *Bacillus velezensis* FZB42, maize, induced systemic resistance, miRNA, degradome sequencing

## Abstract

*Bacillus velezensis* FZB42 is able to activate induced systemic resistance (ISR) to enhance plant defense response against pathogen infections. Though the roles of microRNAs (miRNAs) in *Bacillus*-triggered ISR have been reported in *Arabidopsis*, the maize miRNAs responsible for the *Bacillus*-activated ISR process have not been discovered. To explore the maize miRNAs involved in ISR, maize miRNAs in response to FZB42 (ISR activating), FZB42△*sfp*△*alss* (deficient in triggering ISR), and a control for 12 h were sequenced. A total of 146 known miRNAs belonging to 30 miRNA families and 217 novel miRNAs were identified. Four miRNAs specifically repressed in FZB42-treatment were selected as candidate ISR-associated miRNAs. All of them contained at least one defense response-related cis-element, suggesting their potential roles in activating the ISR process. Interestingly, three of the four candidate ISR-associated miRNAs belong to the conserved miR169 family, which has previously been confirmed to play roles in abiotic stress response. Moreover, 52 mRNAs were predicted as potential targets of these candidate ISR-associated miRNAs through TargetFinder software and degradome sequencing. Gene Ontology (GO) and network analyses of target genes showed that these differentially expressed miRNA might participate in the ISR process by regulating nuclear factor Y transcription factor. This study is helpful in better understanding the regulatory roles of maize miRNAs in the *Bacillus*-activated ISR process.

## 1. Introduction

Plant miRNAs with a length of 21–24 nt are non-coding RNA molecules that regulate target mRNA either through transcriptional or translational repression [1]. Plant miRNA precursors are firstly transcribed by RNA polymerase II and then processed by Dicer-like DCL endonucleases to generate a miRNA/miRNA * duplex. The duplex contains a guide strand (mature miRNA) and a passenger strand (miRNA *) [2]. Plant methyltransferase HUA ENHANCER1 (HEN1) stabilizes the miRNA/miRNA * duplex through 2′-*O*-methylation. The mature miRNA is separated from the duplex and loaded into an Argonaute (AGO) protein to form the RNA-induced silencing complex (RISC). The RISC is able to cleave target mRNAs which have complement sequences to guide the strand [2]. Some mature plant miRNAs can regulate target genes at protein levels instead of at post-transcriptional levels [3].

Plant miRNAs play a part in the development and responses to biotic stresses and the interaction process between plants and beneficial microbes [4,5,6]. For example, the expression level of miR172c has been shown to increase upon rhizobia infection, and the overexpression of miR172c has resulted in increased rhizobia infection and improved nodulation, suggesting that miR172c functions as a key regulator during bean–*Rhizobium* symbiosis [7]. During the symbiosis process, plant miR2111 undergoes shoot-to-root translocation and regulates some key symbiosis suppressors in roots to balance infection and nodulation events [8]. MiR171h and miR396 from *Medicago truncatula* control symbiosis with arbuscular mycorrhizal (AM) fungi by targeting nodulation signaling pathway 2 and growth regulating factor, respectively [9,10]. A few studies have reported the role of miRNAs in *Bacillus*–plant interaction. *Bacillus* spp., a class of typical plant growth promoting rhizobacteria (PGPR), are famous for their heat-resistant spores [11]. The interaction between plants and *Bacillus* is a complicated process. Plant roots initially secrete some specific chemical signals like L-malic acid to attract *Bacillus* spp. [12]. Then, *Bacillus* spp. migrates toward and adheres to the surface of roots. After colonization, *Bacillus* spp. can utilize the carbohydrates and amino acids released by plant roots. They also benefit plants by producing phytostimulators like indole-3-acetic acid (IAA), enhancing the availability of nutrients, producing antibiotic substances to inhibit the growth of pathogens, and triggering induced systemic resistance (ISR) [13,14]. The interaction between plants and *Bacillus* is mutually beneficial. Several plant miRNAs have been reported to participate in this interaction process. For instance, the expression level of *Arabidopsis* miR846 is reduced in response to *Bacillus velezensis* FZB42, the suppression of miR846 induces the target jacalin lectin family genes and triggers ISR via a JA (jasmonic acid)-dependent signaling pathway, further resulting in an enhanced defense response [15]. MiR825/miR825* from *Arabidopsis thaliana* is also suppressed in response to *Bacillus cereus* AR156, and then activates ISR by targeting ubiquitin-protein ligases and leucine-rich repeat resistance genes [16].

Though the roles of miRNAs in *Bacillus*-triggered ISR have been reported in *Arabidopsis*, an investigation on food crops has been limited. Maize (*Zea mays*) is one of the three major grain crops in the world. To better understand the function of maize miRNAs in the ISR-activating process, the commercial strain *B. velezensis* FZB42, which can effectively colonize in the roots of maize, was chosen [17]. In our previous study, we confirmed that the mutant *B. velezensis* FZB42△*sfp*△*alss*, deficient in the production of lipopeptides and 2,3-butanediol, lost the ability to trigger ISR in *Arabidopsis* [15]. To explore maize miRNAs involved in ISR, maize leaves in response to FZB42 (ISR activating), FZB42△*sfp*△*alss* (deficient in triggering ISR), and a control were analyzed by Illumina Hiseq deep sequencing and degradome sequencing. This study will help us to better understand the FZB42-induced maize ISR at the miRNA level.

## 2. Results

### 2.1. Global miRNA Profile Analysis

**Figure 1:**
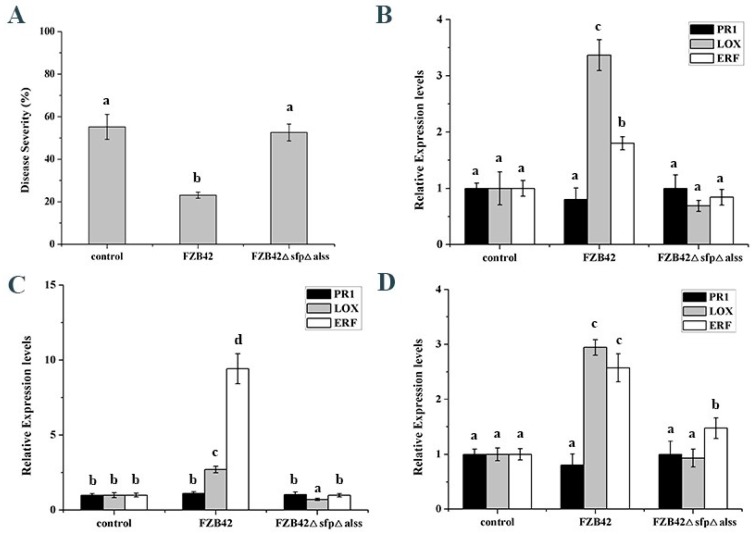
*Bacillus velezensis* FZB42 activates induced systemic resistance (ISR) in maize against *Bipolaris maydis*. (**A**) Disease severity of maize against *B. maydis* after inoculating with FZB42, FZB42△*sfp*△*alss* mutant, and a control. The roots of maize at the four-leaf stage were inoculated with *B. velezensis* FZB42, FZB42△*sfp*△*alss* mutant, or sterilized distilled water. Twenty-four hours later, the maize leaves were sprayed with *B. maydis* conidia. After challenging for seven days, the disease index was measured. The expression levels are shown of the defense-related genes in maize leaves after inoculation with FZB42, FZB42△*sfp*△*alss* mutant and the control for 6 h (**B**), 12 h (**C**) and 24 h (**D**). *PR1* (pathogenesis-related protein), *LOX* (lipoxygenases) and *ERF* (ethylene response factor) are the marker genes of the salicylic acid-, jasmonic acid- and ethylene-dependent signaling pathways, respectively. The expression levels of defense-related genes were analyzed with qRT-PCR and normalized to *GAPDH* and *Actin*. Each treatment contained 12 plants, and the experiment was repeated three times. Different letters indicate statistical differences between treatments (One-way ANOVA; *p* < 0.05).

The roots of maize at the four-leaf stage were first inoculated with *B. velezensis* FZB42, FZB42△*sfp*△*alss,* or a control, and then the maize leaves were challenge-inoculated with *Bipolaris maydis*. The results showed that *B. velezensis* FZB42 reduced the southern corn leaf blight caused by *B. maydis*, whereas the mutant FZB42△*sfp*△*alss*, deficient in the production of lipopeptides and 2,3-butanediol, lost the ability to enhance plant defense resistance (Figure 1A and Figure S1). *PR1*, *LOX* and *ERF* are the marker genes of the salicylic acid-, jasmonic acid-, and ethylene-dependent signaling pathways, respectively [18]. The expression levels of *LOX* and *ERF* in maize leaves were induced after inoculation with FZB42 for 6, 12, and 24 h (Figure 1B–D). These results indicated that FZB42 could trigger induced systemic resistance (ISR) in maize, while the mutant FZB42△*sfp*△*alss* failed to enhance maize defense responses.

**Figure 2:**
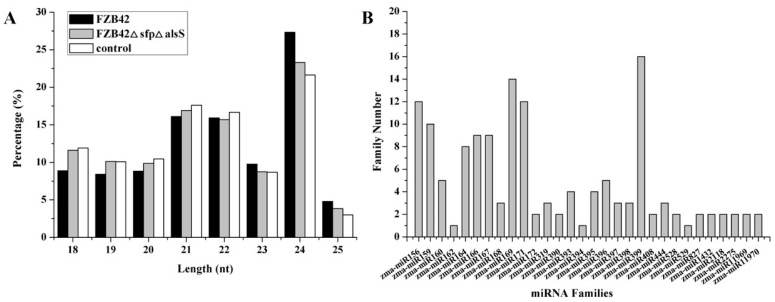
Lengths distribution of small RNAs (**A**) and the abundance of the miRNA family (**B**) from maize leaves inoculated with *B. velezensis* FZB42, FZB42 △*sfp*△*alss* mutant, and the control.

To study the role of miRNAs involved in the ISR-activating process, small RNA libraries from maize leaves inoculated with FZB42, FZB42△*sfp*△*alss* and a control for 12 h were constructed and sequenced. After removing the low quality and contaminated reads, 3,357,929, 3,741,777 and 2,159,692 clean reads, on average, were obtained from the FZB42-treated, FZB42△*sfp*△*alss*-treated, and control samples, respectively (Table S1). These clean reads were then further analyzed for their length distribution. More than 75% of the clean reads had a length ranging from 20 to 24 nt, and the small RNA (sRNA) with a length of 24 nt was the most abundant group (Figure 2A).

### 2.2. Identification of miRNAs in Maize

The small RNA sequences from nine libraries were mapped to the *Zea mays* B73 genome, and the miRBase database release 21 (http://www.mirbase.org/) for miRNA identification. Sequence alignment analysis identified 146 known maize miRNAs belonging to 30 miRNA families in total (Figure 2B and Table S2). The number of each miRNA family member varied from one to sixteen, a peak of the zma-miR399 family with sixteen members was observed in the abundance analysis of miRNA families, followed by the zma-miR169 family with fourteen members. The miREvo and mirdeep2 software were used to predict novel miRNA by exploring the hairpin structure of miRNA precursors. After removing the known miRNAs, ribosomal RNA (rRNA), transfer RNA (tRNA), small nuclear RNA (snRNA) and small nucleolar RNA (snoRNA), 217 novel miRNAs were identified (Table S3). The length of novel miRNA precursors ranged from 58 to 247 nt.

### 2.3. Differentially Expressed miRNAs Responsive to Bacillus

**Figure 3:**
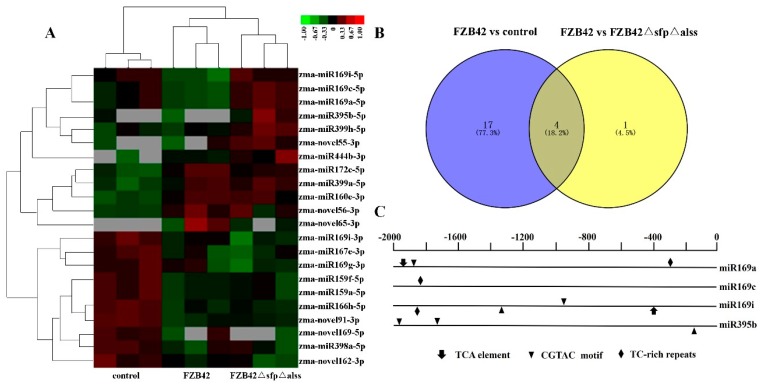
Differentially expressed maize miRNAs responsive to *Bacillus* spp. (**A**) Heatmap of differentially expressed miRNAs in maize leaves inoculated with FZB42, FZB42△*sfp*△*alss*, and the control. (**B**) Venn diagram of differentially expressed miRNAs between (FZB42 versus control) and (FZB42 versus FZB42△*sfp*△*alss*). (**C**) Cis-elements analysis of differentially expressed maize miRNAs. Cis-elements in the promoter region of primary miRNAs (pri-miRNAs) were analyzed with PlantCARE. The cis-elements distributed on the sense strand and reverse strand are shown above and below the black lines, respectively. TCA element, cis-element involved in SA responsiveness; CGTCA motif, cis-element involved methyl jasmonate (MeJA) responsiveness; TC-rich repeats, cis-element involved in defense and stress responsiveness.

Twenty-two miRNAs including sixteen known miRNAs and six novel miRNAs were differentially expressed in maize leaves inoculated with FZB42, FZB42△*sfp*△*alss,* and the control (Table S4). The cluster analysis of these differentially expressed miRNAs showed clearly defined groups for the FZB42-treated, FZB42△*sfp*△*alss*-treated, and control samples, indicating the consistency between biological repeats (Figure 3A). *B. velezensis* FZB42 could trigger ISR to enhance defense resistance in maize, while the mutant FZB42△*sfp*△*alss* lost this ability. Hence, we speculated that ISR-associated maize miRNAs would be differentially expressed in both (FZB42 versus FZB42△*sfp*△*alss*) and (FZB42 versus control) combinations. To evaluate the roles of maize miRNAs involved in ISR, the overlapping parts like zma-miR169a-5p, zma-miR169c-5p, zma-miR169i-5p, and zma-miR395b-5p were selected as candidate ISR-associated miRNAs (Figure 3B). To confirm the expression levels of ISR-associated miRNAs, these four miRNAs were analyzed by stem-loop qRT-PCR (Figure S2). A similar expression pattern was observed with the sequence data, suggesting that the data were reliable and suitable for further analysis. Furthermore, the promoter sequences of the candidate ISR-associated miRNAs were also analyzed with PlantCARE. three types of cis-elements including the TCA element (involved in SA responsiveness), CGTCA motif (involved in methyl jasmonate [MeJA] responsiveness) and TC-rich repeats (involved in defense and stress responsiveness) associated with defense response were focused. The promoters of the four ISR-associated miRNAs contained at least one defense response-related cis-element (Figure 3C). Additionally, three types of defense-related cis-elements were both detected in the promotion of zma-miR169a and zma-miR169i. These results suggested that these miRNAs may participate in the ISR-activating process.

### 2.4. Target Prediction of Differentially Expressed miRNAs

To further explore the regulation functions of miRNAs, maize leaves inoculated with FZB42, FZB42△*sfp*△*alss*, and the control for 12 h were collected and analyzed by degradome sequencing. After removing three adaptors and mapping to *Zea mays* genome, 3,787,305, 3,639,110 and 3,472,813 unique transcript mapped reads were obtained from the FZB42-treated, FZB42△*sfp*△*alss*-treated and control samples, respectively (Table S5). In this study, 1479 mRNAs were predicted as potential targets of the 76 known maize miRNAs and 29 novel miRNAs based on Targetfinder software and degradome sequencing (Table S6). According to the abundance of tags at the cleavage site, target mRNAs were divided into five categories. In Categories 0 and 1, the highest abundance of degradome tags was both found at the cleavage site, only one peak existed in Category 0, and more than one peak existed in category 1; in Categories 2 and 3, the abundances of tags at the cleavage site was lower than the highest abundance, and their abundances were, respectively, higher than or lower than the median abundance; the rest of degradome tags belonged to Category 4. A total of 1479 target mRNAs were obtained, including 399, 52, 467, 41 and 520 mRNAs belonging to Categories 0, 1, 2, 3 and 4, respectively (Table S7). Most of the targets of novel miRNAs were classified into Category 4.

**Figure 4:**
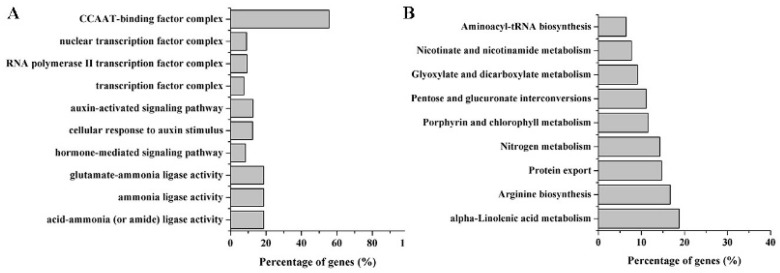
Gene Ontology (GO) term enrichment (**A**) and Kyoto Encyclopedia of Genes and Genomes (KEGG) pathway enrichment (**B**) of target genes of differentially expressed miRNAs. The x-axis is the percentage of enriched target genes to all genes in the category or pathway.

Furthermore, the abundance tags at the cleavage sites of these 1479 mRNAs among the FZB42-treated, FZB42△*sfp*△*alss*-treated and control samples were compared. The same parts between (FZB42 versus FZB42△*sfp*△*alss*) and (FZB42 versus control) combinations were selected out and submitted to the Gene Ontology (GO) and Kyoto Encyclopedia of Genes and Genomes (KEGG) pathway databases. The GO analysis showed that these targets were enriched into acid–ammonia ligase activity, ammonia ligase activity, and glutamate–ammonia ligase activity in the molecular function category (Figure 4A). In the biological process and cellular component categories, the GO terms were enriched into auxin-activated processes and transcription factor complexes, respectively (Figure 4A). In the KEGG pathway analysis, these targets participated in alpha-linoleic acid metabolism, arginine biosynthesis, protein export, nitrogen metabolism, porphyrin and chlorophyll II metabolism, pentose and glucuronate interconversions, glyoxylate and dicarboxylate metabolism, nicotinate and nicotinamide metabolism, and aminoacyl-tRNA biosynthesis (Figure 4B).

**Figure 5:**
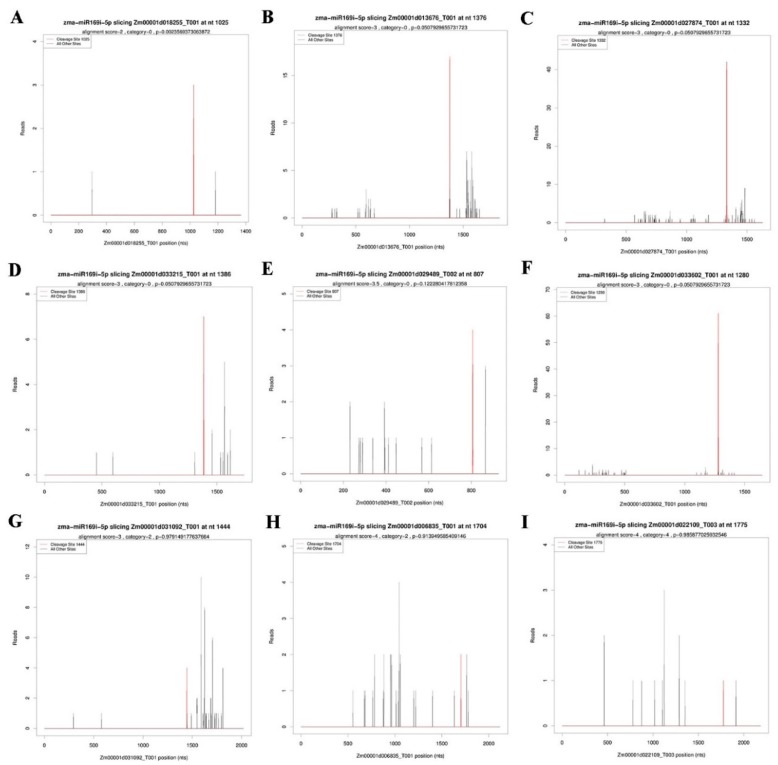
Target identification of zma-miR169i-5p by degradome sequencing. (**A**) Zm00001d018255_T001; (**B**) Zm00001d013676_T001; (**C**) Zm00001d027874_T001; (**D**) Zm00001d033215_T001; (**E**) Zm00001d029489_T002; (**F**) Zm00001d033602_T001; (**G**) Zm00001d031092_T001; (**H**) Zm00001d006835_T001; and (**I**) Zm00001d022109_T003.

**Figure 6:**
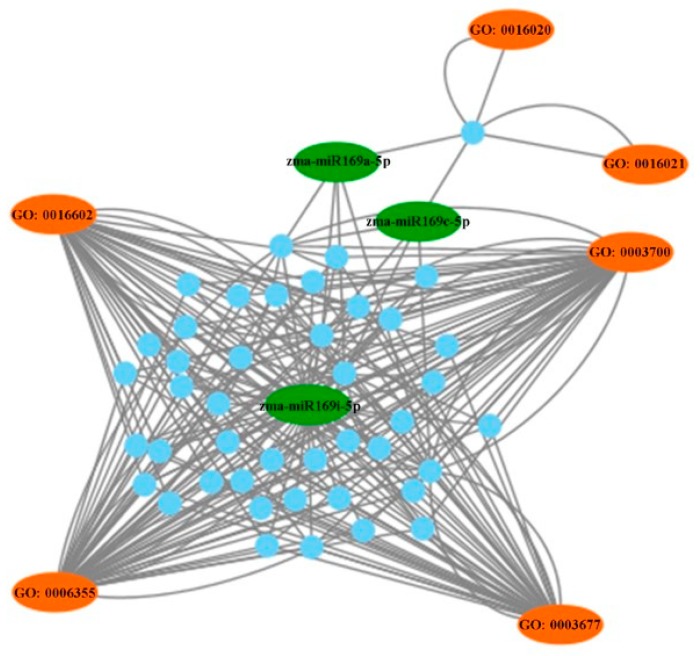
The potential regulatory network of differentially expressed miRNAs in the induced systemic resistance activating process. The green oval represents the down-regulated miRNAs in response to FZB42 at 12 h; the blue dot represents target genes; the orange oval represents the GO term; GO: 0,003,677 represents DNA-binding; GO: 0003700 represents DNA-binding transcription factor activity; GO: 0006355 represents the DNA-templated regulation of transcription; and GO: 0016602 represents the CCAAT-binding factor complex.

The target prediction of candidate ISR-associated miRNAs is conducive to better understanding their regulatory mechanisms involved in ISR. In total, 52 mRNAs were predicted as potential targets of the four ISR-associated miRNAs (Table S8 and Figure 5). No target mRNA of zma-miR395b-5p was identified by degradome sequencing, while zma-miR169i-5p could target 42 mRNAs. Furthermore, the network of the three ISR-associated miRNAs (zma-miR169a-5p, zma-miR169c-5p and zma-miR169i-5p, all belonging to the miR169 family) and their target mRNAs were constructed (Figure 6). The network showed that these target genes mainly participated in DNA-binding, DNA-binding transcription factor activity, DNA-templated regulation of transcription, and CCAAT-binding factor complex.

## 3. Discussion

Plants exhibit an enhanced resistance against some pathogens when the roots are inoculated with beneficial microbes. This phenomenon is known as induced systemic resistance (ISR) [11]. Though ISR shows a similarity with systemic acquired resistance (SAR) in enhancing defense responses, there are some distinctions between them. SAR is often triggered by pathogens and is accompanied by increased salicylic acid (SA) content [19]. However, ISR can be activated by non-pathogenic microbes like plant growth-promoting rhizobacteria (PGPR) and is dependent on the jasmonic acid (JA)- and ethylene (ETH)-signaling pathways [20]. Many miRNAs have been confirmed to play important roles in the SAR process. Ath-miR393 was the first identified miRNA responsible for triggering the SAR process [5]. It opened a door to study the functions of plant miRNAs in SAR. MiR398 targets *CSD1* and *CSD2* (Cu/Zn superoxide dismutase genes) to control reactive oxygen species (ROS) production during pathogen infections [21]. MiR472, miR482 and miR2109 can suppress resistance (R) genes to regulate defense responses [22,23,24]. MiR160a, miR398 and miR733 function as regulators of callose deposition and result in an enhanced defense resistance against *Pseudomonas syringae* DC3000 [25]. The overexpression of miR398, miR400 or miR844 all alters plant resistance to pathogens [26,27]. Since ISR differs from SAR in stimulating inducers and signal transduction, different miRNAs may participate in ISR and SAR. Though Ath-miR846 and Ath-miR825 have been reported to regulate *Bacillus*-induced ISR, they belong to non-conserved miRNAs which exist only in limited plant species [15,16]. There may be other conserved miRNAs in the ISR process.

Bacterial components like flagella and lipopolysaccharides (LPS) can activate ISR. Cyclic lipopeptides and volatile organic compounds (2,3-butanediol) produced by *Bacillus* have been confirmed as inducers of ISR [28,29]. Here, maize miRNAs in response to FZB42 (ISR activating), FZB42△*sfp*△*alss* (deficient in triggering ISR) and the control were analyzed to better understand the roles of miRNAs in ISR. In total, 146 known miRNAs and 217 novel miRNAs were identified. Four miRNAs (zma-miR169a-5p, zma-miR169c-5p, zma-miR169i-5p and zma-miR395b-5p) that were specially repressed in FZB42-treatment were selected as candidate ISR-associated miRNAs. All of them contained at least one cis-element associated with defense response, whereas zma-miR169a and zma-miR169i had the most abundant defense-related cis-elements. In addition, three of the four candidate ISR-associated miRNAs belonged to the miR169 family, which is one of the most ancient miRNA families existing in various plants, like *Arabidopsis*, rice and maize. This family had been confirmed to play roles in abiotic stress responses, like salt, drought and nitrogen stress. In *Arabidopsis*, the overexpression of miR169a is more sensitive to drought stress by increasing leaf water loss [30]. The Ath-miR169a expression in roots and shoots is both down-regulated by nitrogen starvation [31]. In maize, the expressions of zma-miR169 and its target Nuclear Factor Y subunit A (NF-YA) genes are also responsive to drought stress [32]. In addition to its function as a regulator in abiotic stresses, miR169 could also participate in plant immunity. For example, *Arabidopsis* CLAVATA1 (CLV1) and CLAVATA2 (CLV2) receptors contribute to *Ralstonia solanacearum* pathogenicity through the miR169-dependent pathway [33]. Osa-miR169 acts as a negative regulator in rice immunity against the blast fungus *Magnaporthe oryzae* by repressing the expression of NF-YA genes [34]. Moreover, the expression levels of ma-miR169a and ma-miR169b are consistent with the resistance degree of the banana cultivars to *Fusarium oxysporum f*. sp. *Cubense* tropical race 4 [35]. In this study, the expression levels of zma-miR169a, zma-miR169c and zma-miR169i were all repressed in response to *B. velezensis* FZB42 inoculation, suggesting their potential roles in induced systemic resistance (ISR)-activating process.

The target prediction of miRNAs is conducive to better understanding their regulatory mechanisms. A total of 52 mRNAs were predicted as potential targets of zma-miR169a, zma-miR169c and zma-miR169i. The GO and network analyses showed that these target genes mainly participated in DNA-binding, DNA-binding transcription factor activity, the DNA-templated regulation of transcription, and the CCAAT-binding factor complex. Transcription factors control gene expression at the transcription level through DNA-binding domains [36]. According to the characteristics of the DNA-binding domain, transcription factors have been classified into several groups, like APETALA2/ethylene response factor (AP2/ERF), NAM-ATAF1/2-CUC2 (NAC), WRKY and Nuclear factor Y (NY-F). NF-Y transcription factors, also called heme-associated proteins (HAPs), and CCAAT-binding factors are present in all eukaryotes [37]. Based on the presence of conserved domains, NF-Y families are divided into three subfamilies: NF-YA, NF-YB (Nuclear Factor Y subunit B) and NF-YC (Nuclear Factor Y subunit C) [38]. In plants, NF-Ys control plant-specific pathways like drought resistance, nitrogen nutrition and symbiotic plant–microbe interactions [39,40]. Several studies have addressed their potential function in plant defense response. The overexpression of *OsHAP2E* for a CCAAT-binding factor confers resistance to *Cucumber mosaic virus* and *Rice necrosis mosaic virus* [41]. *MtNF-YA1*, previously identified as a key regulator of rhizobial symbiosis establishment, is also a determinant of *Medicago truncatula* susceptibility toward a root pathogen [42]. In summary, our results showed that some members in the miR169 family might regulate NF-Y transcription factors to activate the ISR process.

## 4. Methods

### 4.1. Microbial Strains, Plant and Growth Conditions

*B. velezensis* FZB42 and mutant FZB42△*sfp*△*alss* were cultured in a Luria-Bertani (LB) medium by shaking at 200 rpm at 37 °C overnight. Bacterial cells were then collected by centrifugation and resuspended in sterilized distilled water with a final concentration of 10^8^ colony-forming units (CFU)/mL.

*Bipolaris maydis* was cultured on a niblet culture under 12/12 h at 28 °C for 10 days to produce conidia; the conidia suspensions were then filtered through gauze, washed with sterilized distilled water, and adjusted to the final concentration of 10^5^ CFU/mL with a hemocytometer.

The seeds of maize B73 inbred lines were obtained from the Maize Genetics Cooperation Stock Center (http://maizecoop.cropsci.uiuc.edu/). Maize seeds were firstly sterilized with 70% ethanol for 1 min and washed three times with sterilized distilled water. The washed seeds were germinated at 28 °C for 7 days and then transported to sterilized soil. After growing for three weeks, the roots of maize were inoculated with 50 mL of *B. velezensis* FZB42 or mutant FZB42△*sfp*△*alss* cell suspensions. Sterilized distilled water was used as the control. After inoculation with FZB42, mutant FZB42△*sfp*△*alss,* or the control for 24 h, the maize leaves were sprayed with *B. maydis* conidia. Seven days later, the disease index was measured. Each treatment contained 12 plants, and the experiment was repeated three times.

### 4.2. Construction and Analysis of Small RNA Libraries

After inoculated with FZB42, FZB42△*sfp*△*alss* mutant, or the control for 12 h, five maize per sample were harvested and sent to LC Bio (Hangzhou, China) for miRNA library construction and sequencing. For the library construction, total RNA was first extracted from nine samples. The small RNA was ligated with 3′ adaptor and 5′ adaptor, transcribed to complementary DNA (cDNA), and followed by PCR amplification. The PCR products were run on PAGE (polyacrylamide gel) and purified. The purified PCR was sequenced on Illumina Hiseq platform. Raw reads were cleaned by removing low quality and contaminated reads, and they were then analyzed for their length distribution. The raw data were deposited in the National Center for Biotechnology Information (NCBI) database Short Read Archive (SRA) (https://www.ncbi.nlm.nih.gov/sra) under accession number PRJNA575329. Clean reads were mapped to the *Zea mays* B73 genome and miRBase database release 21 (http://www.mirbase.org/) for miRNA identification. The miREvo (http://evolution.sysu.edu.cn/software/mirevo.htm) and mirdeep2 software (https://www.mdc-berlin.de) were used to predict novel miRNA by exploring the hairpin structure of miRNA precursors.

### 4.3. Identification of Induced Systemic Resistance-Associated miRNAs

To identify the induced systemic resistance-associated miRNAs, the miRNA expression levels were normalized by transcript per million and then analyzed with DEGseq method. The miRNAs with a *p*-value < 0.05 were selected as differentially expressed miRNAs. The common, differentially expressed miRNAs in (FZB42 versus control) and (FZB42 versus △*sfp*△*alss*) were referred as ISR-associated miRNAs.

### 4.4. Analysis of miRNA Promoter

The promoter sequences of maize pri-miRNAs were obtained as following [43]. In brief, if the pri-miRNA and its upstream gene transcribed in the same direction and their distance was <2.4 kb, the region between the site 400 bp downstream of the upstream gene and pri-miRNA was used. If the pri-miRNA and its upstream gene transcribed in the opposite direction and the distance between them was >4 kb, the sequence from pri-miRNA to their middle point was selected. Otherwise, the 2 kb sequence upstream of pri-miRNA was obtained. These pri-miRNA promoters were finally analyzed with PlantCARE (http://bioinformatics.psb.ugent.be/webtools/plantcare).

### 4.5. cDNA Library Construction for Degradome Sequencing

After being inoculated with FZB42, FZB42△*sfp*△*alss* mutant, or the control for 12 h, the total RNA from maize leaves were isolated and purified using poly-T oligo-attached magnetic beads. The 3′ cleavage products of the mRNA were ligated with 5′ adapters, transcribed to cDNA, and followed by PCR amplification. Finally, the 50 bp single-end sequencing on an Illumina Hiseq 2500 (LC Bio, Hangzhou, China) were performed. The raw data were deposited in the NCBI database SRA (https://www.ncbi.nlm.nih.gov/sra) under accession number PRJNA575525.

### 4.6. Prediction of miRNA Targets

TargetFinder software (http://www.bioit.org.cn/ao/targetfinder.htm) and degradome sequencing were used to predict the target genes of differentially expressed miRNAs. The predicted targets were then submitted to the GO (http://www.blast2go.com) and KEGG pathway (https://www.genome.jp/kegg/kaas/) databases to explore the regulation function involved in induced systemic resistance. The network was constructed through Cytoscape software [44]. The differentially expressed miRNAs, target genes, and GO terms were set as nodes, and their relationships were set as edges.

### 4.7. qRT-PCR Analysis

After being inoculated with FZB42, FZB42△*sfp*△*alss* mutant or the control for 6, 12 and 24 h, the maize leaves were harvested and used for RNA isolation. qRT-PCR analysis was performed with a 7300 real-time PCR system (Applied Biosystems, Foster City, CA, USA). The *GAPDH* and *Actin* genes were used as internal references.

The expression levels of candidate ISR-associated miRNAs were verified with stem-loop qRT-PCR. Total RNA was reverse transcribed to first-strand cDNA with a stem-loop reverse transcription primer, and then performed with a 7300 real-time PCR system (Applied Biosystems, Foster City, CA, USA). The 18s rRNA was used as internal references. The primer sequences of these genes are listed in Table S9. The expression levels of marker genes and miRNAs were calculated according to the 2^−△△*c*t^ relative quantification method. Each sample contained three biological replicates and was repeated three times.

### 4.8. Statistical Analysis

The data were analyzed by Fisher’s least-significant difference test (*p* < 0.05) with SPSS software (SPSS Inc. Chicago, IL, USA).

## 5. Conclusions

A total of 146 known miRNAs belonging to 30 miRNA families and 217 novel miRNAs were identified. Four miRNAs, including zma-miR169a-5p, zma-miR169c-5p, zma-miR169i-5p and zma-miR395b-5p, that were specifically repressed in FZB42-treament were selected as candidate ISR-associated miRNAs. Three types of defense response-related cis-elements were predicted in their promoters. Zma-miR169a and zma-miR169i had the most abundant defense-related cis-elements. These candidate ISR-associated miRNAs might regulate NF-Y transcription factors to activate the ISR process in maize.

## Supplementary Materials

Supplementary materials can be found at https://www.mdpi.com/1422-0067/20/20/5057/s1.
