# Supplementary material for: Identification of miRNAs Involved in Bacillus velezensis FZB42-Activated Induced Systemic Resistance in Maize
Source: Int J Mol Sci. 2019 Oct 12;20(20):5057. doi: 10.3390/ijms20205057 (PMC6829523; doi:10.3390/ijms20205057)
Supplement: Supplementary file 1 [file ijms-20-05057-s001.zip › Figure S2.docx]

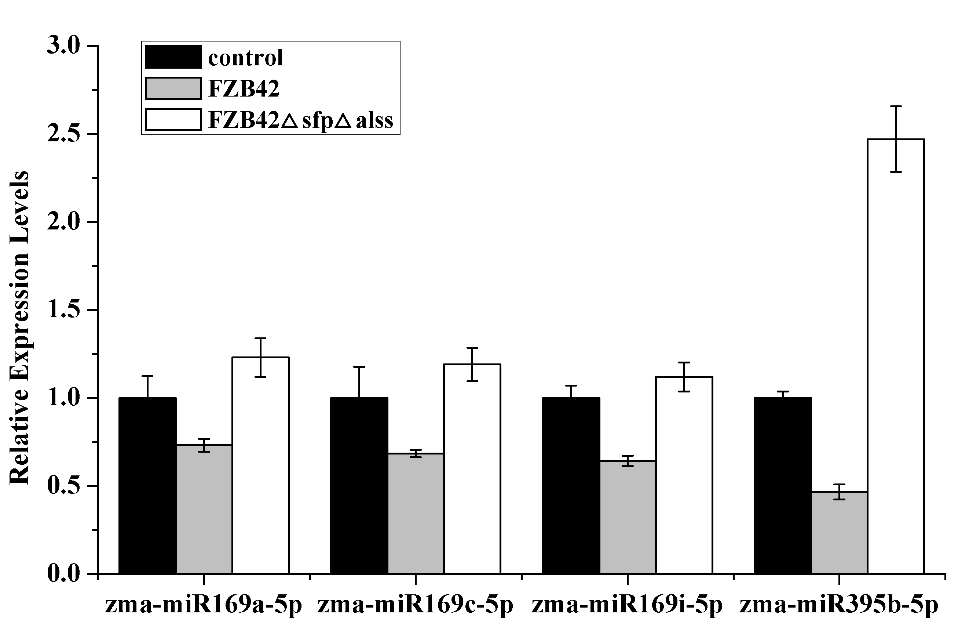


Figure S2 Expression levels of candidate ISR-associated miRNAs in response to *Bacillus velezensis* FZB42, FZB42△*sfp*△*alss* and control
