# Supplementary material for: Identification of miRNAs Involved in Bacillus velezensis FZB42-Activated Induced Systemic Resistance in Maize
Source: Int J Mol Sci. 2019 Oct 12;20(20):5057. doi: 10.3390/ijms20205057 (PMC6829523; doi:10.3390/ijms20205057)
Supplement: Supplementary file 1 [file ijms-20-05057-s001.zip › Table S1.docx]

Table S1 Summary of small RNA sequencing

| Sample | Raw reads | 3ADT&length filter | Junk reads | Rfam | mRNA | Repeats | Clean reads |
| --- | --- | --- | --- | --- | --- | --- | --- |
| Control-1 | 12231198 | 8334961 | 1304 | 507200 | 499959 | 45051 | 2921749 |
| Control-2 | 14292267 | 12107487 | 753 | 290675 | 251089 | 26589 | 1660399 |
| Control-3 | 16846807 | 14380933 | 1064 | 309545 | 279191 | 23725 | 1896928 |
| FZB42-1 | 12109046 | 4942599 | 6528 | 773435 | 870362 | 31903 | 560400 |
| FZB42-2 | 16258408 | 14017827 | 783 | 305849 | 275980 | 32795 | 1674374 |
| FZB42-3 | 14184848 | 10409571 | 1092 | 546212 | 465572 | 56986 | 2795413 |
| FZB42△*sfp*△*alss*-1 | 18958746 | 12141226 | 1571 | 1030202 | 862141 | 119289 | 5989699 |
| FZB42△*sfp*△*alss*-2 | 14128979 | 12449847 | 564 | 218477 | 214607 | 19611 | 1260215 |
| FZB42△*sfp*△*alss*-3 | 25559142 | 19047891 | 4935 | 743212 | 875255 | 42804 | 4975417 |
