# Supplementary material for: Identification of miRNAs Involved in Bacillus velezensis FZB42-Activated Induced Systemic Resistance in Maize
Source: Int J Mol Sci. 2019 Oct 12;20(20):5057. doi: 10.3390/ijms20205057 (PMC6829523; doi:10.3390/ijms20205057)
Supplement: Supplementary file 1 [file ijms-20-05057-s001.zip › Table S2.docx]

Table S2 Family information of known miRNAs identified from maize leaves

| miRNA family | miRNA name | miRNA family | miRNA name |
| --- | --- | --- | --- |
| zma-miR156 | zma-miR156a-5p |  | zma-miR166j-3p |
|  | zma-miR156a-3p |  | zma-miR166l-3p |
|  | zma-miR156b-3p |  | zma-miR166m-5p |
|  | zma-miR156d-3p | zma-miR167 | zma-miR167a-5p |
|  | zma-miR156e-3p |  | zma-miR167c-3p |
|  | zma-miR156h-3p |  | zma-miR167d-3p |
|  | zma-miR156i-3p |  | zma-miR167e-5p |
|  | zma-miR156j-5p |  | zma-miR167e-3p |
|  | zma-miR156j-3p |  | zma-miR167f-3p |
|  | zma-miR156k-5p |  | zma-miR167g-3p |
|  | zma-miR156k-3p |  | zma-miR167h-3p |
|  | zma-miR156l-3p |  | zma-miR167j-3p |
| zma-miR159 | zma-miR159a-5p | zma-miR168 | zma-miR168a-5p |
|  | zma-miR159a-3p |  | zma-miR168a-3p |
|  | zma-miR159b-3p |  | zma-miR168b-3p |
|  | zma-miR159c-3p | zma-miR169 | zma-miR169a-5p |
|  | zma-miR159f-5p |  | zma-miR169a-3p |
|  | zma-miR159f-3p |  | zma-miR169b-3p |
|  | zma-miR159h-3p |  | zma-miR169c-5p |
|  | zma-miR159i-3p |  | zma-miR169c-3p |
|  | zma-miR159j-3p |  | zma-miR169f-5p |
|  | zma-miR159k-3p |  | zma-miR169f-3p |
| zma-miR160 | zma-miR160a-5p |  | zma-miR169g-3p |
|  | zma-miR160b-3p |  | zma-miR169h-5p |
|  | zma-miR160e-3p |  | zma-miR169i-5p |
|  | zma-miR160f-5p |  | zma-miR169i-3p |
|  | zma-miR160f-3p |  | zma-miR169m-5p |
| zma-miR162 | zma-miR162-3p |  | zma-miR169m-3p |
| zma-miR164 | zma-miR164a-5p |  | zma-miR169r-3p |
|  | zma-miR164b-3p | zma-miR171 | zma-miR171a-3p |
|  | zma-miR164c-3p |  | zma-miR171c-3p |
|  | zma-miR164d-3p |  | zma-miR171d-5p |
|  | zma-miR164e-5p |  | zma-miR171d-3p |
|  | zma-miR164e-3p |  | zma-miR171f-3p |
|  | zma-miR164f-5p |  | zma-miR171g-5p |
|  | zma-miR164h-5p |  | zma-miR171g-3p |
| zma-miR166 | zma-miR166a-5p |  | zma-miR171h-5p |
|  | zma-miR166a-3p |  | zma-miR171h-3p |
|  | zma-miR166b-5p |  | zma-miR171i-3p |
|  | zma-miR166c-5p |  | zma-miR171j-3p |
|  | zma-miR166g-5p |  | zma-miR171l-3p |
|  | zma-miR166h-5p | zma-miR172 | zma-miR172a |
|  | zma-miR172c-5p |  | zma-miR399d-5p |
| zma-miR319 | zma-miR319a-5p |  | zma-miR399d-3p |
|  | zma-miR319a-3p |  | zma-miR399e-5p |
|  | zma-miR319c-5p |  | zma-miR399e-3p |
| zma-miR390 | zma-miR390a-5p |  | zma-miR399f-5p |
|  | zma-miR390a-3p |  | zma-miR399f-3p |
| zma-miR393 | zma-miR393a-5p |  | zma-miR399g-5p |
|  | zma-miR393a-3p |  | zma-miR399g-3p |
|  | zma-miR393b-5p |  | zma-miR399h-5p |
|  | zma-miR393c-3p |  | zma-miR399i-5p |
| zma-miR394 | zma-miR394a-5p |  | zma-miR399j-5p |
| zma-miR395 | zma-miR395a-5p | zma-miR408 | zma-miR408a |
|  | zma-miR395a-3p |  | zma-miR408b-5p |
|  | zma-miR395b-5p | zma-miR444 | zma-miR444a |
|  | zma-miR395e-5p |  | zma-miR444b-5p |
| zma-miR396 | zma-miR396a-5p |  | zma-miR444b-3p |
|  | zma-miR396a-3p | zma-miR528 | zma-miR528a-5p |
|  | zma-miR396c |  | zma-miR528a-3p |
|  | zma-miR396e-5p | zma-miR529 | zma-miR529-5p |
|  | zma-miR396f-3p | zma-miR827 | zma-miR827-5p |
| zma-miR397 | zma-miR397a-5p |  | zma-miR827-3p |
|  | zma-miR397b-5p | zma-miR1432 | zma-miR1432-5p |
|  | zma-miR397b-3p |  | zma-miR1432-3p |
| zma-miR398 | zma-miR398a-5p | zma-miR2118 | zma-miR2118b |
|  | zma-miR398a-3p |  | zma-miR2118d |
|  | zma-miR398b-5p | zma-miR2275 | zma-miR2275a-3p |
| zma-miR399 | zma-miR399a-5p |  | zma-miR2275b-3p |
|  | zma-miR399a-3p | zma-miR11969 | zma-miR11969-5p |
|  | zma-miR399b-5p |  | zma-miR11969-3p |
|  | zma-miR399b-3p | zma-miR11970 | zma-miR11970-5p |
|  | zma-miR399c-5p |  | zma-miR11970-3p |
