# Supplementary material for: Identification of miRNAs Involved in Bacillus velezensis FZB42-Activated Induced Systemic Resistance in Maize
Source: Int J Mol Sci. 2019 Oct 12;20(20):5057. doi: 10.3390/ijms20205057 (PMC6829523; doi:10.3390/ijms20205057)
Supplement: Supplementary file 1 [file ijms-20-05057-s001.zip › Table S4.docx]

Table S4 Differentially expressed miRNAs in maize leaves inoculated with FZB42, FZB42△*sfp*△*alss* and control

| miRNA Name | P value | FZB42 | FZB42△*sfp*△*alss* | control |
| --- | --- | --- | --- | --- |
| zma-miR159a-5p | 1.33E-02 | 38 | 40 | 72 |
| zma-miR159f-5p | 1.33E-02 | 38 | 40 | 72 |
| zma-miR160e-3p | 4.10E-02 | 160 | 160 | 11 |
| zma-miR166h-5p | 2.66E-05 | 20 | 21 | 86 |
| zma-miR167e-3p | 2.66E-02 | 111 | 109 | 151 |
| zma-miR169a-5p | 6.89E-03 | 74 | 98 | 87 |
| zma-miR169c-5p | 6.89E-03 | 74 | 98 | 87 |
| zma-miR169g-3p | 2.77E-02 | 173 | 149 | 195 |
| Zma-miR169i-5p | 4.52E-02 | 139 | 176 | 174 |
| zma-miR169i-3p | 2.37E-03 | 205 | 173 | 257 |
| zma-miR172c-5p | 3.43E-03 | 58 | 48 | 17 |
| zma-miR395b-5p | 2.13E-02 | 1 | 8 | 2 |
| zma-miR398a-5p | 4.45E-02 | 364 | 379 | 560 |
| zma-miR399a-5p | 9.10E-03 | 519 | 584 | 327 |
| zma-miR399h-5p | 2.18E-02 | 23 | 40 | 22 |
| zma-miR444b-3P | 4.00E-02 | 6 | 10 | 1 |
| zma-novel55-3p | 2.33E-02 | 4 | 12 | 2 |
| zma-novel56-3p | 4.72E-02 | 35 | 30 | 18 |
| zma-novel65-3p | 2.25E-02 | 7 | 3 | 0 |
| zma-novel91-3p | 4.81E-05 | 472 | 209 | 230 |
| zma-novel162-3p | 3.66E-02 | 199 | 176 | 243 |
| zma-novel169-5p | 4.72E-02 | 9 | 4 | 1 |
