# Supplementary material for: Identification of miRNAs Involved in Bacillus velezensis FZB42-Activated Induced Systemic Resistance in Maize
Source: Int J Mol Sci. 2019 Oct 12;20(20):5057. doi: 10.3390/ijms20205057 (PMC6829523; doi:10.3390/ijms20205057)
Supplement: Supplementary file 1 [file ijms-20-05057-s001.zip › Table S5.docx]

Table S5 Summary of degradome sequencing

| Sample | control | FZB42 | FZB42△*sfp*△*alss* |
| --- | --- | --- | --- |
| Total unique raw reads | 4704242 (100%) | 4387207 (100%) | 4401138 (100%) |
| Unique mappable reads | 4679609 (99.48%) | 4363169 (99.45%) | 4378494 (99.49%) |
| Unique transcript mapped reads | 3787305 (80.51%) | 3639110 (82.95%) | 3472813 (78.91%) |
| Total number of input transcript | 131612 (100%) | 131612 (100%) | 131612 (100%) |
| Number of covered transcript | 117719 (89.44%) | 118006 (89.66%) | 118669 (90.17%) |
