# Supplementary material for: Identification of miRNAs Involved in Bacillus velezensis FZB42-Activated Induced Systemic Resistance in Maize
Source: Int J Mol Sci. 2019 Oct 12;20(20):5057. doi: 10.3390/ijms20205057 (PMC6829523; doi:10.3390/ijms20205057)
Supplement: Supplementary file 1 [file ijms-20-05057-s001.zip › Table S9.docx]

Table S9 Primers used in this study

| Name | Primer sequence (5’-3’) |
| --- | --- |
| zm-PR1-F | ACTACGTGGACCCGCACAAC |
| zm-PR1-R | CGGAGTGGATCAGCTGGCAGTC |
| zm-LOX-F | AGGAGTTTGGACGGGAGATT |
| zm-LOX-R | CCGTACTTGCTCGGGTCA |
| zmERF-F | GGTCAATTTCCGGGACGAAGC |
| zmERF-R | CGTTGCCACAAGCAGTTGGAGTAG |
| GADPH-F | CTTCGGCATTGTTGAGGGTTTG |
| GADPH-R | TCCTTGGCTGAGGGTCCGTC |
| zma-miR169a-5p-RT | GTCGTATCCAGTGCAGGGTCCGAGGTATTCGCACTGGATACGACTCGGCA |
| zma-miR169a-5p -F | ACACTCCAGCTGGGCAGCCAAGGATGACT |
| zma-miR169c-5p -RT | GTCGTATCCAGTGCAGGGTCCGAGGTATTCGCACTGGATACGACCCGGCA |
| zma-miR169c-5p -F | ACACTCCAGCTGGGCAGCCAAGGATGACT |
| zma-miR169i-5p -RT | GTCGTATCCAGTGCAGGGTCCGAGGTATTCGCACTGGATACGACCAGGCA |
| zma-miR169i-5p -F | ACACTCCAGCTGGGTAGCCAAGGATGACT |
| zma-miR395b--5p-RT | GTCGTATCCAGTGCAGGGTCCGAGGTATTCGCACTGGATACGACTTGTGA |
| zma-miR395b-5p-F | ACACTCCAGCTGGGGTTCCCTACAAGCACT |
| stem-loop-R | GTGCAGGGTCCGAGGTATTC |
| 18s-F | CCATCCCTCCGTAGTTAGCTTCT |
| 18s-R | CCTGTCGGCCAAGGCTATATAC |
