# Supplementary figures and images for: Identification of miRNAs Involved in Bacillus velezensis FZB42-Activated Induced Systemic Resistance in Maize
Source: Int J Mol Sci. 2019 Oct 12;20(20):5057. doi: 10.3390/ijms20205057 (PMC6829523; doi:10.3390/ijms20205057)

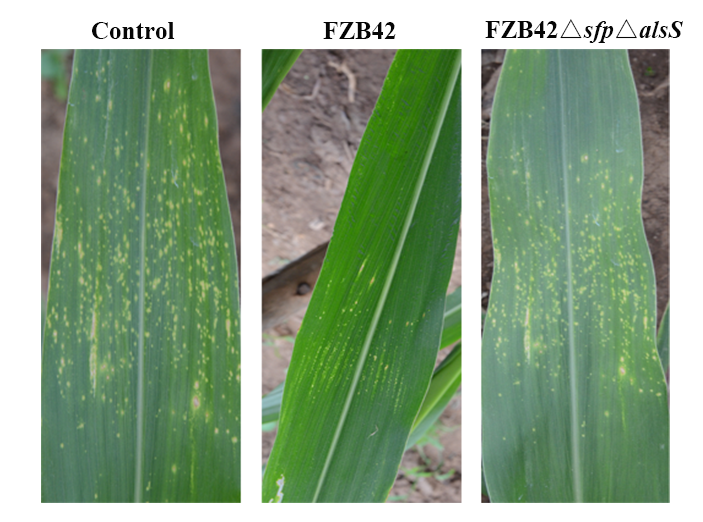


Figure S1 *Bacillus velezensis* FZB42 enhances maize defense response against *Bipolaris maydis*.

Supplement: Supplementary file 1 [file ijms-20-05057-s001.zip › Figure S1.docx]
